# Intracellular Toxic AGEs (TAGE) Triggers Numerous Types of Cell Damage

**DOI:** 10.3390/biom11030387

**Published:** 2021-03-05

**Authors:** Masayoshi Takeuchi, Akiko Sakasai-Sakai, Takanobu Takata, Jun-ichi Takino, Yoshiki Koriyama, Chigusa Kikuchi, Ayako Furukawa, Kentaro Nagamine, Takamitsu Hori, Tamihide Matsunaga

**Affiliations:** 1 Department of Advanced Medicine, Medical Research Institute, Kanazawa Medical University, 1-1 Daigaku, Uchinada-machi, Ishikawa 920-0293, Japan; asakasai@kanazawa-med.ac.jp (A.S.-S.); takajjjj@kanazawa-med.ac.jp (T.T.); 2 Department of Biochemistry, Faculty of Pharmaceutical Sciences, Hiroshima International University, 5-1-1 Hirokoshingai, Kure, Hiroshima 737-0112, Japan; j-takino@hirokoku-u.ac.jp (J.-i.T.); hori@hirokoku-u.ac.jp (T.H.); 3 Graduate School and Faculty of Pharmaceutical Sciences, Suzuka University of Medical Science, 3500-3 Minamitamagaki, Suzuka, Mie 513-8670, Japan; koriyama@suzuka-u.ac.jp (Y.K.); furukawa@suzuka-u.ac.jp (A.F.); 4 Department of Clinical Pharmacy, Graduate School of Pharmaceutical Sciences, Nagoya City University, 3-1 Tanabe-dori, Mizuho-ku, Nagoya 467-8603, Japan; kikuchi@phar.nagoya-cu.ac.jp (C.K.); tmatsu@phar.nagoya-cu.ac.jp (T.M.); 5 Department of Clinical Nutrition, Faculty of Health Sciences, Hiroshima International University, 5-1-1 Hirokoshingai, Kure, Hiroshima 737-0112, Japan; k-nagamine@hirokoku-u.ac.jp

**Keywords:** advanced glycation end-products (AGEs), toxic AGEs (TAGE), Alzheimer’s disease (AD), non-alcoholic steatohepatitis (NASH), cardiovascular disease (CVD), lifestyle-related diseases (LSRD)

## Abstract

The habitual intake of large amounts of sugar, which has been implicated in the onset/progression of lifestyle-related diseases (LSRD), induces the excessive production of glyceraldehyde (GA), an intermediate of sugar metabolism, in neuronal cells, hepatocytes, and cardiomyocytes. Reactions between GA and intracellular proteins produce toxic advanced glycation end-products (toxic AGEs, TAGE), the accumulation of which contributes to various diseases, such as Alzheimer’s disease, non-alcoholic steatohepatitis, and cardiovascular disease. The cellular leakage of TAGE affects the surrounding cells via the receptor for AGEs (RAGE), thereby promoting the onset/progression of LSRD. We demonstrated that the intracellular accumulation of TAGE triggered numerous cellular disorders, and also that TAGE leaked into the extracellular space, thereby increasing extracellular TAGE levels in circulating fluids. Intracellular signaling and the production of reactive oxygen species are affected by extracellular TAGE and RAGE interactions, which, in turn, facilitate the intracellular generation of TAGE, all of which may contribute to the pathological changes observed in LSRD. In this review, we discuss the relationships between intracellular TAGE levels and numerous types of cell damage. The novel concept of the “TAGE theory” is expected to open new perspectives for research into LSRD.

## 1. Introduction

Diabetes mellitus (DM), one of the lifestyle-related diseases (LSRD), is regarded as one of greatest public health challenges of the 21st century. Increases have been reported annually in the number of patients with DM, which may result in life-changing complications. The global prevalence of DM was estimated to be 463 million individuals in 2019, and is expected to increase to 578 million by 2030 and 700 million by 2045 [1]. Furthermore, the prevalence of impaired glucose tolerance (IGT), which increases the risk of DM, was estimated to be 374 million worldwide in 2019, and is expected to increase to 454 million by 2030 and 548 million by 2045 [1]. Previous studies have reported that the chronic intake of beverages and processed foods containing excessive amounts of sugar (i.e., sucrose and high-fructose corn syrup, HFCS) not only causes obesity, metabolic syndrome (MetS), and DM, but also contributes to the onset/progression of non-alcoholic fatty liver disease (NAFLD)/non-alcoholic steatohepatitis (NASH), cardiovascular disease (CVD), and Alzheimer’s disease (AD); however, the underlying mechanisms responsible for these effects currently remain unknown [2,3,4,5].

Advanced glycation end-products (AGEs) are produced under hyperglycemic states in the body via a non-enzymatic glycation reaction (the Maillard reaction) between the ketone or aldehyde groups of reducing sugars and α-/ε-amino groups or guanidino groups of proteins [6,7]. The sugars/carbonyl compounds involved in this reaction influence the types of AGEs produced. Yellow-brown fluorescence and the formation of protein cross-links with and between amino or guanidino groups (i.e., pentosidine, argpyrimidine, methylglyoxal (MGO)-lysine dimer (MOLD), glyoxal (GO)-lysine dimer (GOLD), crosslines) were originally considered to be the characteristics of AGEs. However, the term AGEs now comprises a wide range of advanced glycation products, such as Nε-(carboxymethyl)lysine (CML), Nε-(carboxyethyl)lysine (CEL), MGO-derived hydroimidazolone (MG-H1), GO-derived hydroimidazolone (G-H1), and pyrraline, which are colorless, do not fluoresce, or do not form cross-links [6,7,8,9,10]. Sugar concentrations, the turnover rates of chemically modified targets, and time have all been shown to influence the in vivo generation of AGEs. Although elevated concentrations of glucose were previously suggested to markedly affect the Maillard reaction, it is now recognized as one of the least reactive sugars in biological organisms. In addition to the extracellular generation of AGEs, researchers are now beginning to focus on their rapid intracellular production from intracellular precursors, including trioses (i.e., glyceraldehyde (GA)) and dicarbonyl compounds (i.e., MGO, GO, and 3-deoxyglucosone (3-DG)). Since the structures of AGEs in vivo markedly vary and the reactions required for their production are complex, the structures of only a few AGEs have been elucidated to date [6,7,9,10]. The structures of cytotoxic AGEs remain largely unknown.

AGEs derived from GA, a glucose and fructose metabolic intermediate, exhibit strong cytotoxicity and, thus, are called toxic AGEs (TAGE). The accumulation of TAGE has been implicated in the pathogenesis of AD [11,12,13], NAFLD/NASH [14,15,16,17], CVD [18,19,20], and DM and diabetic microvascular complications [21,22,23]. We recently demonstrated that intracellular TAGE generation/accumulation induced not only neuronal cell damage [24,25], but also hepatocellular damage [26,27,28], pancreatic ductal epithelial cell damage [29], cardiomyocyte pulsation arrest and cell death [30], and myoblast cell death [31]. Therefore, the accumulation of TAGE in cells induces cell damage, resulting in their extracellular leakage into the blood, and thus, increased levels in circulating fluids [32]. Interactions between extracellular TAGE and the receptor for AGEs (RAGE) alter intracellular signaling, gene expression, and the release of pro-inflammatory molecules, and also induce the generation of reactive oxygen species (ROS) in numerous types of cells [23], all of which may contribute to the pathological changes observed in LSRD. The close relationships between the generation/accumulation of intracellular TAGE and numerous types of cell damage are discussed herein.

## 2. Generation Routes for Various AGEs in the Human Body

**Figure 1:**
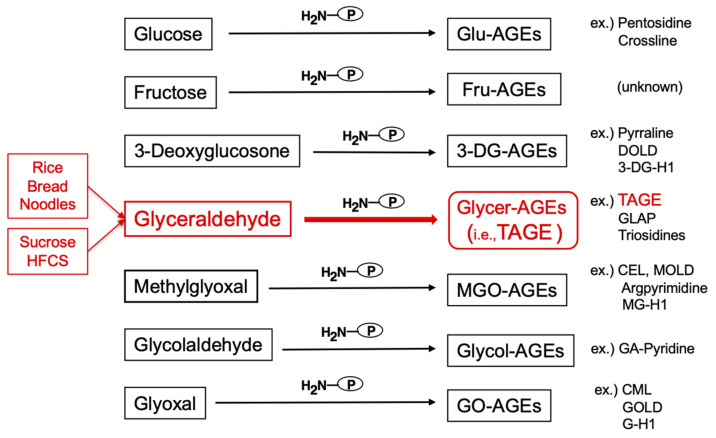
Various routes for the in vivo production of advanced glycation end-products (AGEs). Reducing sugars, including glucose, fructose, and glyceraldehyde, react non-enzymatically with the amino/guanidino groups of proteins, resulting in the formation of reversible Schiff bases and Amadori products/Heyns products. Further complex reactions involving these early glycation products, such as rearrangement, dehydration, and condensation reactions, lead to irreversibly cross-linked, heterogeneous fluorescent derivatives, named AGEs. Glu-AGEs: glucose-derived AGEs; Fru-AGEs: fructose-derived AGEs; 3-DG-AGEs: 3-deoxyglucosone-derived AGEs; Glycer-AGEs: glyceraldehyde-derived AGEs; TAGE: toxic AGEs; MGO-AGEs: methylglyoxal-derived AGEs; Glycol-AGEs: glycolaldehyde-derived AGEs; GO-AGEs: glyoxal-derived AGEs; DOLD: 3-deoxyglucosone-lysine dimer; 3-DG-H1: 3-deoxyglucosone-derived hydroimidazolone; GLAP: glyceraldehyde-derived pyridinium; CEL: Nε-(carboxyethyl)lysine; MOLD: methylglyoxal-lysine dimer; MG-H1: methylglyoxal-derived hydroimidazolone 1; GA-pyridine: glycolaldehyde-derived pyridine; CML: Nε-(carboxymethyl)lysine; GOLD: glyoxal-lysine dimer; G-H1; glyoxal-derived hydroimidazolone 1; HFCS: high-fructose corn syrup; P-NH_2_: free amino residue of a protein.

AGEs are generated and subsequently accumulate in a number of tissues with normal aging, and these processes are markedly accelerated in DM [6,33]. We previously reported the involvement of α-hydroxyaldehydes (i.e., GA and glycolaldehyde), dicarbonyl compounds (i.e., MGO, GO, and 3-DG), fructose, and glucose in protein glycation [6,34]. Previous studies have detected seven immunochemically distinct classes of AGEs (Figure 1) and CML in the serum samples of hemodialysis patients with diabetic nephropathy (HD-DN) [6,34]. Accordingly, the in vivo generation of AGEs has been suggested to occur via a process involving sugar metabolism pathways, sugar autoxidation, and the Maillard reaction. Glycer-AGEs derived from GA, a triose sugar intermediate of fructose and glucose metabolism, exhibit strong cytotoxicity [11]; therefore, we proposed the novel concept of TAGE [35]. Differences have been reported between the epitope recognized by the anti-TAGE antibody and GA-derived AGE structures; i.e., 3-hydroxy-5-hydroxymethyl-pyridinium (GLAP) compound [36] and triosidines [37]. We also identified differences between the anti-TAGE antibody and antibodies against well-defined AGEs as well as those generated from reducing sugar/carbonyl molecules with unknown structures (International patent application for anti-TAGE antibody: PCT/JP2019/34195).

TAGE are produced from digested starch, the main component of rice, bread, and noodles, as well as metabolites of the sugars (sucrose and HFCS) added to beverages and processed foods, and fluctuations in TAGE levels in the human body are closely associated with dietary habits (Figure 1).

## 3. Formation/Metabolism of MGO

MGO is mainly formed by the fragmentation of the glycolytic intermediates glyceraldehyde 3-phosphate (GA3P) and dihydroxyacetone phosphate (DHAP) [8,38,39,40], whereas the primary sources of production of GO are lipid peroxidation and sugar degradation [40]. MGO and GO have both been identified as very potent glycating agents. MGO is a major precursor for the generation of AGEs, and its interaction with arginine, which modifies this amino acid, leads to the formation of specific MGO-AGEs, including the methylglyoxal-derived hydroimidazolone, MG-H1. 

**Figure 2:**
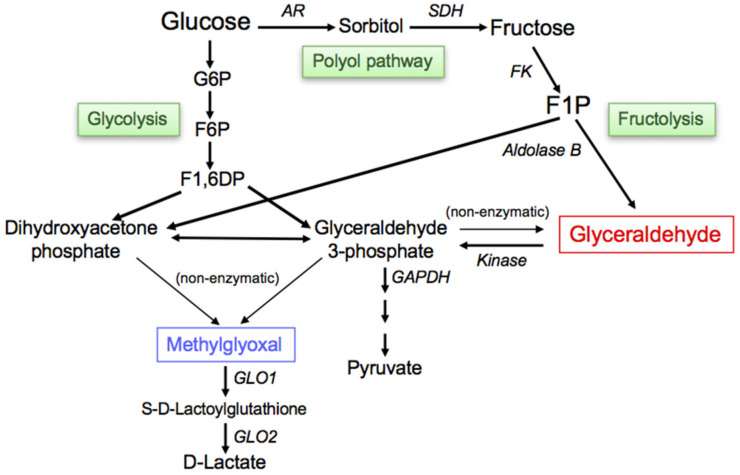
Routes for the in vivo production of glyceraldehyde (GA)/methylglyoxal (MGO). The glycolytic intermediate glyceraldehyde 3-phosphate (GA3P) is generally catabolized (glycolysis) by the enzyme GA3P dehydrogenase (GAPDH). However, decreases in the enzymatic activity of GAPDH result in the intracellular accumulation of GA3P. As a consequence, GA3P is metabolized via an alternative pathway, which increases the concentration of GA. Fructokinase phosphorylates fructose to fructose 1-phosphate (F1P), which is then converted into dihydroxyacetone phosphate (DHAP) and GA by aldolase B (fructolysis). MGO is mainly produced as a byproduct of non-enzymatic reactions with GA3P or DHAP during glycolysis. The most effective MGO metabolic pathway is the glyoxalase system, which converts MGO to D-lactate. G6P: glucose 6-phosphate; F6P: fructose 6-phosphate; F1,6DP: fructose 1,6-diphosphate; AR: aldose reductase; SDH: sorbitol dehydrogenase; FK: fructokinase; GLO1: glyoxalase 1; GLO2: glyoxalase 2.

Multiple pathways have been shown to contribute to the metabolism of MGO in order to prevent abnormally high levels of this reactive compound. The most effective MGO and GO metabolic pathway is the glyoxalase system, comprising glyoxalase 1 (GLO1) and glyoxalase 2 (GLO2), which converts MGO to D-lactate via the intermediate product S-D-lactoylglutathione in the presence of reduced glutathione [8,38,39,40] (Figure 2). 

## 4. The Generation/Accumulation of GA, a Precursor of TAGE

GA is generated in cells via the following three pathways: fructolysis, glycolysis, and the polyol pathway [41] (Figure 2), and promotes the generation of TAGE in intracellular proteins. 

### 4.1. Fructolysis

The fructolytic pathway is essential for the metabolism of fructose, particularly in the liver. Fructose is quickly phosphorylated to fructose 1-phosphate (F1P) by fructokinase, and is then cleaved by aldolase B to produce DHAP and GA. A previous study reported F1P cleavage (aldolase) activity in the human brain and heart [42].

### 4.2. Glycolysis

The glycolytic pathway plays a crucial role in the metabolism of glucose. GA3P, which is an intermediate of this pathway, is metabolized by GA3P dehydrogenase (GAPDH). In the presence of reduced GAPDH activity, accumulating GA3P is shifted to an alternative metabolic route and is then non-enzymatically dephosphorylated and degraded to GA [43]. 

### 4.3. Polyol Pathway

The polyol pathway converts glucose to sorbitol via aldose reductase and then sorbitol to fructose via sorbitol dehydrogenase under hyperglycemic conditions. Fructose is metabolized via fructolysis to GA. Increases in intracellular glucose levels under hyperglycemic conditions have been shown to activate the polyol pathway in insulin-independent tissues, including the liver, brain, and heart, which generates fructose [43].

These findings suggest that TAGE are generated intracellularly and may then spread throughout the body. 

## 5. RAGE

Different types of AGE-binding proteins have been identified. Receptor-dependent/independent mechanisms contribute to AGE-induced cellular dysfunction and tissue damage. Receptor-mediated biological reactions by AGEs have been extensively examined. RAGE, a pattern recognition receptor that belongs to the immunoglobulin G superfamily, has been characterized in the most detail, and ligand binding at its extracellular domain has been shown to initiate a complex intracellular signaling cascade. Amyloid β (Aβ), S100/calgranulin, and high mobility group box-1 are endogenous ligands of RAGE [44]. A previous study demonstrated that interactions between AGEs and RAGE elicited cellular responses via distinct pathways through the activation of nuclear factor kappa B (NF-κB) and Ras-related C3 botulinum toxin substrate-1 (Rac-1)/cdc42, resulting in cytokine production, cell migration, phagocytosis, maturation, and polarization [45]. Other receptors of AGEs with opposite functions to RAGE, such as AGE-R1/-R2/-R3 and the scavenger receptor family, play important roles in the homeostasis of AGEs [46]. Other than RAGE, it currently remains unclear whether TAGE bind to these receptors. Previous studies reported variations in AGE receptor expression between different types of cells and tissues that were affected by metabolic changes [47,48]. Different cell types (i.e., neurons, microglia, hepatocytes, hepatic stellate cells (HSC), cardiomyocytes, fibroblasts, endothelial cells (EC), and pericytes) are known to express RAGE [46]. 

We previously attempted to identify which of the seven distinct classes of AGEs and CML bind to RAGE using a surface-plasmon resonance analysis with purified human RAGE proteins, which produced apparent dissociation constants of 0.36 μM for TAGE and 1.35 μM for Glycol-AGEs. A radiolabeled ligand-binding assay with RAGE-expressing COS-7 cells (a monkey kidney-derived cell line) revealed similar association kinetics [49,50]. However, response signals were not obtained for Glu-AGEs, Fru-AGEs, 3-DG-AGEs, MGO-AGEs, GO-AGEs, or CML. Our findings revealed the following: (i) seven distinct classes of AGE structures are present in the blood of patients with HD-DN [6,34]; (ii) the neurotoxic effects of the serum fraction from HD-DN patients containing various AGE structures are completely neutralized by antibodies against TAGE [11]; (iii) TAGE mimic the deleterious effects of AGE-rich serum purified from HD-DN on EC [51]; and (iv) TAGE are more cytotoxic than other AGEs due to their stronger binding affinities to RAGE [49,50]. 

## 6. Cytotoxicity of TAGE in the Brain

It has been suggested that nutritional factors influence not only the risk of neurological disorders, but also their rate of progression. The abnormal metabolism of glucose in DM patients increases the risk of neurological disorders, such as AD. AD is the most common cause of dementia in developed countries. It is pathologically characterized by the presence of senile plaques (SP) and neurofibrillary tangles (NFT) at extracellular and intracellular sites. SP consist of the Aβ protein, the deposition of which is regarded as an early and causative event in the pathogenesis of AD. It markedly increases during the progression of AD, leading to the generation of NFT and neuronal cell death [52]. Since the incidence of AD is 2–5-fold higher in DM patients, many studies have investigated whether DM is a clinical risk factor for the onset/progression of AD [53,54]. AGEs are involved in the development of the pathological hallmarks of AD, i.e., the accumulation of Aβ is increased by Aβ glycation, and the formation of NFT from phosphorylated tau is accelerated by glycation of the tau protein [55,56]. 

### 6.1. Localization of TAGE in AD Brains 

TAGE mainly localize to the cell bodies of neurons in the hippocampus and parahippocampal gyrus of AD brains, and are not present in SP or astrocytes [12]. However, Glu-AGEs have been detected in the amyloid cores of SP and in astrocytes. TAGE are primarily localized in the perikarya of neurons and produce a uniform staining pattern, which differs from the dot-like pattern produced by Glu-AGE staining. Moreover, Glu-AGEs have been detected at both intracellular and extracellular sites, whereas TAGE are only present intracellularly, suggesting differences in the mechanisms underlying the neurotoxicity of Glu-AGEs and TAGE [12]. 

### 6.2. Effects of Extracellular TAGE on Neuronal Cells 

We previously provided direct immunochemical evidence for the existence of seven distinct AGE structures and CML within the AGE-modified proteins and peptides that circulate in the sera of HD-DN [6,34]. We investigated the types of AGEs involved in the development of the AD pathology using primary cultured cortical neuronal cells [11]. In an incubation of cortical neurons with seven immunochemically distinct classes of AGEs and CML, TAGE exhibited marked cytotoxicity towards cells, and this toxicity was neutralized by the addition of an anti-TAGE specific antibody. We fractionated serum from normal controls and HD-DN by gel filtration and identified two fractions that contained numerous AGE epitopes. The addition of these two fractions led to the death of cultured neuronal cells, and this cytotoxic effect was completely abrogated by the addition of the anti-TAGE specific antibody, but not antibodies against other AGEs or CML [11]. 

### 6.3. Intracellular TAGE and Neuronal Cell Death

The abnormal metabolism of glucose in DM patients increases the risk of AD. We examined the relationship between impaired cerebral glucose metabolism and the pathogenesis of AD in human neuroblastoma SH-SY5Y cells treated with GA, a precursor of TAGE. The findings obtained revealed the following:

(i) The intracellular accumulation of TAGE was induced in GA-treated SH-SY5Y cells, which, ultimately, led to cell death. The production of TAGE in SH-SY5Y cells was dose-dependently increased by GA [24].
(ii) GAPDH activity was decreased in neuronal cells treated with GA [24]. GAPDH activity was reduced in AD patients [57], and GAPDH played a role in apoptosis in neurodegenerative disorders [58]. We previously reported that TAGE inactivated GAPDH in a neuronal culture system [11]. These findings are indicative of a feed-forward mechanism, with extracellular TAGE-induced GAPDH inactivation further stimulating the generation of intracellular TAGE.
(iii) Aβ42 levels in cerebrospinal fluid (CSF) were significantly lower in AD patients than in age-matched healthy elderly controls, whereas total tau and p-tauT181 levels in CSF were significantly higher in AD patients than in the controls [59,60]. Furthermore, the levels of other AD biomarkers, such as vascular endothelial growth factor (VEGF) [61] and transforming growth factor-β1 (TGF-β1) [62], were higher in the CSF of AD patients. We previously reported that the intracellular generation of TAGE decreased Aβ42 levels and increased total tau and p-tauT181 levels in culture media, and also elevated the intracellular levels of AD biomarkers in SH-SY5Y cells [24].

These findings suggest that intracellular TAGE are general causative agents of the onset/progression of AD. 

### 6.4. Mechanisms by Which Intracellular TAGE Cause Cell Damage in Neuronal Cells

A neuropathological hallmark of AD is the intracellular accumulation of NFT comprising paired helical filaments (PHF) and straight filaments. The microtubule-associated protein tau is the main component of PHF [63,64]. The polymerization of dimers of α- and β-tubulin, which self-assemble, is involved in the construction of microtubules. They function as architectural elements to support the elongated shape of microtubules. 

**Figure 3:**
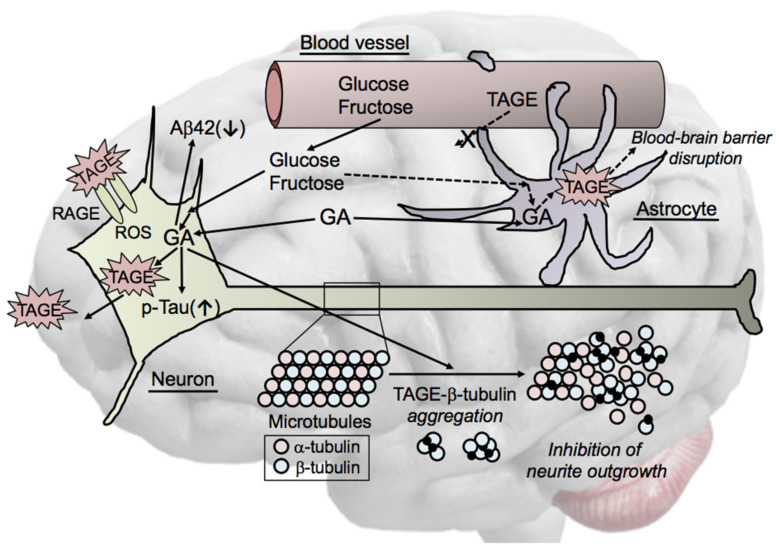
Cytotoxicity of TAGE in neuronal cells. TAGE mainly localize to neuronal cell bodies of neurons in AD brains. GA induces the generation of TAGE and exhibits cytotoxicity towards neuronal cells. Neuronal cell death may induce the extracellular leakage of TAGE, which affects the surrounding cells via the TAGE-RAGE-ROS system. TAGE reduce the concentration of amyloid β1-42 (Aβ42) in culture media and increase tau phosphorylation. β-Tubulin is a target of TAGE-modified proteins. GA induces abnormal β-tubulin aggregation and inhibits neurite outgrowth through the formation of TAGE-β-tubulin. We also hypothesize that TAGE-targeted proteins are involved in GA-induced blood-brain barrier disruption in astrocytes. GA: glyceraldehyde; RAGE: receptor for AGEs; ROS: reactive oxygen species; TAGE: toxic AGEs.

We investigated target proteins for TAGE using two-dimensional gel electrophoresis and mass spectrometry, and the findings obtained demonstrated that β-tubulin was one of the targets of TAGE. The formation of TAGE-β-tubulin, the abnormal aggregation of β-tubulin, and the inhibition of neurite outgrowth were observed in GA-treated SH-SY5Y cells [25]. These findings implicated the formation of TAGE-β-tubulin in the generation of PHF, a component of NFT. They also indicated that the abnormal aggregation of β-tubulin induced by GA prevented β-tubulin from forming normal heterodimers with α-tubulin and inhibited the polymerization and stabilization of microtubules (Figure 3). 

Collectively, these findings suggest that a reduction in the interaction between modified tau and modified tubulin synergistically favors tau and β-tubulin aggregation into PHF, and hence, glycation may promote the formation of PHF in AD. Therefore, TAGE may be general causative agents of the onset of neurodegenerative diseases, such as AD. Although the exact structure of TAGE-β-tubulin, the mechanisms responsible for its formation, and its downstream signaling pathway currently remain unclear, we identified β-tubulin as one of the targets of TAGE [25]. In addition, we demonstrated that GA induced the abnormal aggregation of β-tubulin and inhibited neurite outgrowth. Further studies are needed to elucidate the exact mechanisms underlying GA-induced β-tubulin aggregation.

### 6.5. Cell Signaling of TAGE in Neurons

In addition to neurons, RAGE is expressed by different cell types in the brain, such as EC, astrocytes, and microglia [65]. One of the main mediators of the AGE-related pathogenicity of AD is the activation of RAGE [66], the levels of which were found to be elevated in several cell types in the AD brain [67]. A number of receptors in neural cells other than RAGE bind to AGEs, such as AGE-R2/-R3 [68,69]. Previous studies reported RAGE-dependent inflammatory responses via NF-κB signaling in microglia. The activation of nicotinamide adenine dinucleotide phosphate oxidase (NOX) induced the production of ROS through RAGE, which resulted in the death of EC [70] and neurons [71]. Furthermore, RAGE-dependent signaling in transgenic AD model mice was shown to stimulate inflammatory responses and processes that exacerbated neuronal cell death [72]. However, limited information is currently available on RAGE-dependent cellular signaling pathways in neurons. A RAGE overexpression study using neurons revealed increases in Aβ production, neurotoxicity, and synaptic loss [73]. 

We previously reported that glycolysis was suppressed by GA through the ROS-induced inhibition of hexokinase and GAPDH [24]. Furthermore, GAPDH was found to be inactivated in the brains of AD patients [57,58]. Therefore, although intracellular TAGE proteins are released extracellularly due to cell rupture by TAGE-related cytotoxicity and interact with RAGE and/or AGE-R2/-R3, it currently remains unclear whether TAGE bind to AGE-R2/-R3. We demonstrated that TAGE were mainly present in the neurons of the hippocampus and parahippocampal gyrus and generally localized to the cell bodies of neurons in the brains of AD patients [12]. We also showed that a treatment with GA increased the intracellular generation of TAGE and induced cell dysfunction, including β-tubulin aggregation, tau phosphorylation, and the inhibition of glycolysis [24,25]. 

Further studies are warranted to elucidate the cellular signaling mechanisms underlying TAGE-induced neurotoxicity.

## 7. Cytotoxicity of TAGE in the Liver

The prevalence of MetS is increasing worldwide, and NAFLD, a MetS phenotype, is currently the most common liver disorder. The progression of NAFLD to NASH (the “multiple parallel hits hypothesis”) has been explained by a range of “parallel hits”, including oxidative stress, insulin resistance, genetic and epigenetic factors, environmental elements, nutritional factors, and the gut microflora [74]. The habitual excessive intake of sugar-sweetened beverages (SSB) and processed foods containing large amounts of sugars (i.e., sucrose and HFCS) and dietary AGEs [32], which is characteristic of the modern diet, disturbs the metabolic system in hepatocytes and causes TAGE to be generated/accumulated due to excessive GA production. TAGE are strongly cytotoxic and induce intra-/extracellular damage in hepatocytes [17,26,27,28,46,75,76,77,78].

### 7.1. Intracellular TAGE and Hepatocyte Cell Death 

TAGE have been detected in the hepatocytes of patients with NASH, whereas negligible TAGE levels were found in those with non-alcoholic fatty liver, and no significant differences were observed in CML or Glu-AGE levels between these groups [14,32]. The cytotoxic effects of TAGE have been suggested to contribute to NASH-mediated hepatocyte dysfunction.

(i) The accumulation of TAGE and abnormal heat shock cognate 70 (Hsc70) protein levels were observed in GA-treated human hepatocellular carcinoma (HCC) Hep3B cells without changes in Hsc70 mRNA expression levels [26]. Elevated C-reactive protein (CRP) mRNA levels were reduced to control levels by a pretreatment with aminoguanidine (AG), an inhibitor of the formation of AGEs, which indicated that the intracellular generation and accumulation of TAGE activated inflammatory responses [26].
(ii) Previous studies examined Hep3B cells incubated with GA- or high fructose-containing media and identified heterogeneous nuclear ribonucleoprotein M (hnRNPM) as a TAGE-modified protein [76,77]. hnRNPM, an RNA-binding protein, contributes to many processes in nucleic acid metabolism, such as alternative splicing, mRNA stabilization, and transcriptional and translational regulation. The expression levels of genes associated with extracellular exosome-containing extracellular spaces were altered (up- or down-regulated) by the knockdown of hnRNPM [77].
(iii) Caspase-3 plays a crucial role in apoptosis and has been identified as a target of TAGE-induced modifications in GA-treated HCC HepG2 cells. The cleavage and activation of caspase-3, resulting in protease activity, was previously reported during apoptosis [79]. However, a relationship was observed between increases in TAGE-modified caspase-3 levels and the loss of enzymatic activity, and TAGE-induced modifications also inhibited the cleavage of poly (ADP-ribose) polymerase, which is downstream of caspase-3 in the apoptotic cascade. Furthermore, necrotic-type cell death appeared to be promoted by TAGE-modified caspase-3 [27,78].

These findings suggest that intracellular TAGE contribute to hepatocyte damage.

### 7.2. Mechanisms by Which Intracellular TAGE Induce Cell Death in Hepatocytes

A critical factor in the pathogenesis of NASH is hepatocyte cell death. However, limited information is currently available on the factors responsible for and mechanisms underlying NASH-related cell death. We recently revealed that hepatocyte cell death caused by the intracellular accumulation of TAGE was inhibited by a treatment with N-acetyl-cysteine, a powerful antioxidant [28]. We also demonstrated GA-induced ROS production in cells and increases in the mRNA expression levels of nuclear factor erythroid 2-related factor 2 (Nrf2) and hemeoxygenase-1 in GA-treated cells, indicating GA-induced enhancements in ROS production and the activation of Nrf2-regulated stress response factors [28]. ROS were also shown to increase the expression levels of CRP in hepatocytes [80], suggesting the initiation of inflammatory responses by GA-induced ROS, which is characteristic of NASH. A major source of ROS in various mammalian cells is the mitochondrion [81,82]. The mitochondrial membrane potential was found to be abnormal in GA-treated cells and inhibited in cells pretreated with AG [28]. 

**Figure 4:**
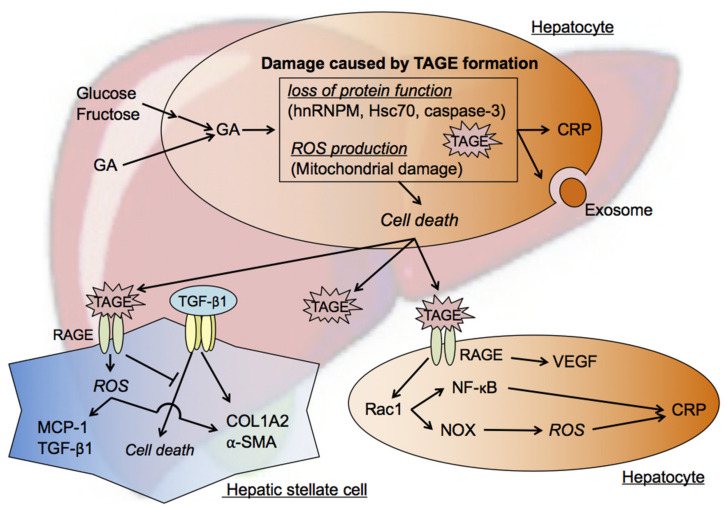
TAGE cytotoxicity against hepatocytes and hepatic stellate cells (HSC). The chronic intake of excessive amounts of sugar-sweetened beverages and processed food products increases the concentration of the sugar metabolite GA in hepatocytes. GA accumulation in these cells promotes TAGE modifications in cellular components. Loss of protein function and mitochondrial membrane abnormalities are associated with the formation of TAGE. ROS are also produced intracellularly and ultimately cause hepatocyte death. Hepatocyte death may cause TAGE-modified proteins to leak from cells, and extracellular TAGE affect the surrounding cells via the TAGE-RAGE axis. The TAGE-RAGE axis exerts inflammatory effects within hepatocytes. HSC are also affected by extracellular TAGE. ROS production and fibrosis are promoted in HSC through the activation of TAGE-RAGE signaling. In addition, TAGE inhibit TGF-β1-induced cell death and help to maintain the number of HSC, resulting in increased extracellular matrix molecule production and, ultimately, fibrosis. GA: glyceraldehyde; TAGE: toxic AGEs; hnRNPM: heterogenous nuclear ribonucleoprotein M; Hsc70: heat shock cognate 70; ROS: reactive oxygen species; CRP: C-reactive protein; RAGE: receptor for AGEs; MCP-1: monocyte chemoattractant protein-1; TGF-β1: transforming growth factor-β1; COL1A2: collagen-type Iα2 chain; α-SMA: α-smooth muscle actin; VEGF: vascular endothelial growth factor; Rac1: Ras-related C3 botulinum toxin substrate 1; NF-κB: nuclear factor kappa B; NOX: nicotinamide adenine dinucleotide phosphate reduced (NADPH) oxidase.

Therefore, ROS were identified for the first time as a direct trigger of cell death due to the intracellular accumulation of TAGE, and novel insights were obtained into the role of TAGE in the pathogenesis of NASH (Figure 4).

### 7.3. Effects of Extracellular TAGE on Hepatocytes

Inflammation in hepatocytes is enhanced by extracellular TAGE through the TAGE-RAGE axis, and this is a feature of the pathology of NASH. Previous studies reported that the expression of CRP was up-regulated in RAGE-expressing Hep3B cells treated with TAGE [46]. Rac-1 mediates the expression of CRP, which is followed by the activation of NF-κB or NOX, both of which are strongly expressed in NASH patients/mice [83,84]. NOX generates ROS, which enhance inflammation in Hep3B cells. In addition to the effects of extracellular TAGE, the accumulation of intracellular TAGE exerts inflammatory effects in hepatocytes [46].

TAGE have been shown to increase VEGF expression levels in Hep3B cells [75]. EC proliferation, migration, and tube formation were found to be significantly enhanced when these cells were incubated in TAGE-treated conditioned medium, indicating increases in the angiogenic potential of Hep3B cells by TAGE-RAGE signaling through the up-regulated expression of VEGF [75]. The TAGE-RAGE signaling pathway in HepG2 cells has also been detected in EC. The TAGE-RAGE axis-mediated, NOX-induced production of ROS has been shown to promote crucial steps in tumor angiogenesis, namely, the proliferation of and tube formation by EC, which are crucial steps in tumor angiogenesis, through the expression of VEGF, which, in turn, was induced via the transcriptional activation of NF-κB and activator protein-1 [51,85,86]. Activation of the TAGE-RAGE axis also induced inflammatory and thrombogenic reactions in EC via the expression of plasminogen activator inhibitor-1, intercellular adhesion molecule-1, and MCP-1 through the production of ROS [23,87,88,89].

These findings suggested the induction of inflammation by TAGE through their intra-/extracellular effects as well as their contribution to the onset/progression of NAFLD/NASH.

### 7.4. Effects of Extracellular TAGE on HSC

Liver fibrosis is characterized by the accumulation of extracellular matrix (ECM) molecules caused by chronic liver damage, including NASH, and its progression induces liver cirrhosis [90]. Under normal conditions, HSC are quiescent and their main function is to store vitamin A [91]. On the other hand, in chronic liver damage, HSC are activated by various cytokines, such as TGF-β1, tumor necrosis factor-α, and platelet-derived growth factors (PDGF), resulting in their differentiation into myofibroblast-like cells and the secretion of a large amount of ECM material, including collagen I [92]. The treatment of human HSC (LI90 cells) with TAGE induced intracellular oxidative stress via the RAGE-NOX-based generation of ROS [93]. Furthermore, the mRNA expression levels of fibrotic genes (i.e., α-smooth muscle actin (α-SMA), TGF-β1, and collagen type lα2) were found to be increased in TAGE-treated cells. The expression levels of MCP-1, which plays a role in inflammation, were also elevated [93]. These findings indicate that TAGE are involved in the onset/progression of hepatic fibrosis because they promote ROS production and HSC activation via RAGE. 

TGF-β1 is a significant activator of HSC [94]. Activated HSC has been shown to participate in the onset/progression of HCC [95,96,97]. We demonstrated that the treatment of human HSC LX-2 cells with TGF-β1 induced apoptosis, while a caspase inhibitor significantly inhibited these effects. TGF-β1-induced apoptosis was suppressed in the presence of TAGE. Although the expression of α-SMA, an indicator of HSC activation, was increased by TGF-β1, its expression level was not altered by a co-treatment with TAGE. Similarly, the expression levels of PDGF receptor β and its ligand PDGF-B, which are also involved in HSC activation and proliferation, were altered by the TGF-β1 treatment, but not by the TAGE co-treatment [98]. Moreover, the mRNA expression level of collagen I per cell was unchanged, whereas the amount of collagen I protein secreted into the culture medium was significantly increased by the TAGE and TGF-β1 co-treatment [98]. These findings indicated that TAGE increased the total production of ECM molecules, such as collagen I, by suppressing apoptosis in activated HSC; i.e., LX-2 cells, after the TGF-β1 treatment. 

Therefore, TAGE may aggravate liver fibrosis in patients with chronic hepatitis, such as NASH (Figure 4).

## 8. Cytotoxicity of TAGE in the Heart

CVD, one of the most significant public health issues of the 21st century, is a LSRD. Postprandial hyperglycemia is related to the onset/progression of diabetic macrovascular disease [99,100]. DM is a major risk factor for CVD morbidity and mortality, and the incidence of CVD is 2–4-fold higher in DM patients than in the general population [101]. Accumulated evidence suggests that postprandial hyperglycemia plays an important role in the pathogenesis of CVD [102,103]. Although CVD is associated with DM [32,104,105], recent studies revealed that the risk of CVD has increased in healthy humans due to lifestyles involving the consumption of abundant amounts of calorie-rich food [106,107]. Relationships between the excessive intake of sugar and risk factors for CVD have been reported in DM patients and healthy humans [108,109,110].

### 8.1. Postprandial Hyperglycemia and TAGE Generation 

TAGE may partly contribute to the increased risk of CVD observed in DM and IGT patients with postprandial hyperglycemia. Collectively, our findings suggest the potential of TAGE as novel predictive/therapeutic targets for the prevention of CVD in patients with DM [23,32].

We previously identified serum TAGE levels as a marker of cumulative postprandial hyperglycemia in Goto-Kakizaki rats, a rat model of DM, and rats treated with nateglinide, a rapid-acting insulin secretagogue [111]. We also observed significant reductions in serum TAGE levels, but not hemoglobin A1c or Glu-AGE levels, in DM patients treated for 12 weeks with acarbose, an α-glucosidase inhibitor [22]. Therefore, the hyperglycemia-induced oxidative stress-mediated inhibition of GAPDH may increase GA levels [112] and subsequently enhance the generation of TAGE during the postprandial period.

### 8.2. Intracellular TAGE and Cardiomyocyte Cell Damage

We recently demonstrated that intracellular TAGE were generated in cardiomyocytes, in which they decreased pulsation rates and induced cell death [30]. Immunohistochemistry was performed and revealed the absence of cells in some areas of GA-treated samples, indicating cell destruction and death. The death of cardiomyocytes may result in heart failure. However, it has not yet been established whether these mechanisms involve the same or different pathways. To clarify whether cardiomyocyte pulsation and/or viability were decreased by TAGE generation, cardiomyocytes were pretreated with AG, which inhibited not only reductions in pulsation and cell viability, but also TAGE generation [30].

### 8.3. Mechanisms by Which Intracellular TAGE Cause Cell Damage in Cardiomyocytes

We previously suggested that intracellular TAGE suppress autophagy. Autophagy is an intracellular degradation process that contributes to the recycling of long-lived, aggregated, or misfolded proteins or even entire organelles [113,114]. The dysregulation of autophagy has been implicated in several diseases, including cardiomyopathy, cancer, and neurodegeneration [115]. We examined the autophagy-related factors LC3-II/LC3-I and p62 [113]. Although intracellular TAGE were clearly shown to exert cytotoxic effects in cell lines from various organs [24,26,27,29,31], our findings revealed the time-dependent down-regulation of LC3-II/LC3-I expression in cardiomyocytes after a treatment with TAGE [30]. Cellular homeostasis is maintained by autophagy, which generally occurs at basal levels [113,115]. 

**Figure 5:**
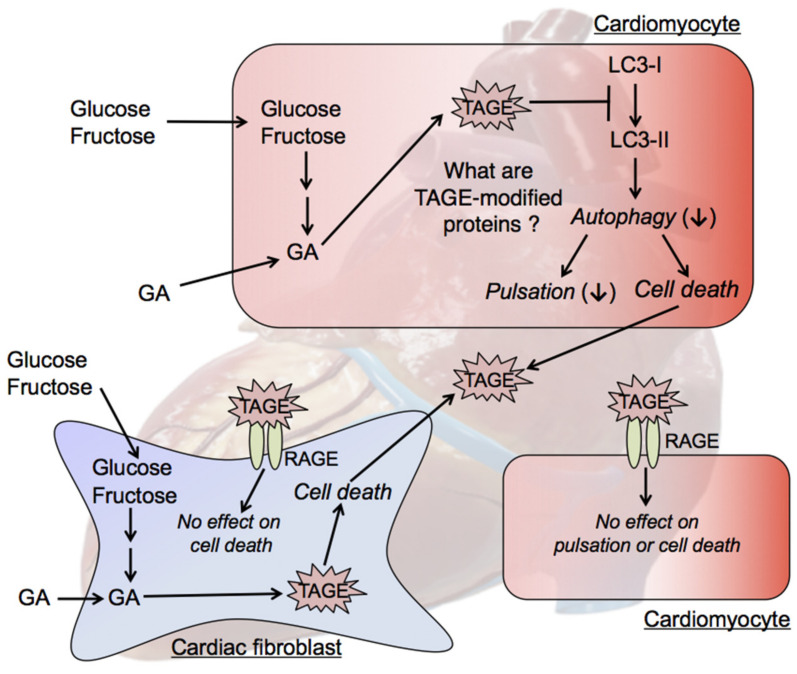
TAGE cytotoxicity against cardiomyocytes and cardiac fibroblasts. GA accumulation in cardiomyocytes leads to TAGE-induced modifications in cellular components, including proteins. TAGE may be released from dead cardiomyocytes; however, extracellular TAGE do not suppress cardiomyocyte pulsation or induce cell death in cardiomyocytes via the TAGE-RAGE axis. The intracellular generation of TAGE in cardiac fibroblasts and their cytotoxicity, and the effects of extracellular TAGE on cardiac fibroblasts have not yet been elucidated. GA: glyceraldehyde; LC3 (MAP1LC3): microtubule-associated protein light chain 3; RAGE: receptor for AGEs; TAGE: toxic AGEs.

Although the TAGE-modified proteins that are generated in cardiomyocytes have not yet been identified, proteins that regulate autophagy-related pathways, such as those involved in the production of LC3-II, may be modified by TAGE. TAGE may be generated intracellularly in human and rat cardiomyocytes and directly induce damage, leading to CVD. Future studies are needed to identify TAGE-modified proteins and clarify the mechanisms by which the generation of TAGE leads to the development of CVD (Figure 5).

### 8.4. Intracellular TAGE and Cardiac Fibroblast Cell Death

We treated cardiac fibroblasts with GA, and found that intracellular TAGE were generated in cardiac fibroblasts and induced cell death. Cardiac fibroblasts are a critical cell population because they are responsible for myocardial ECM homeostasis [116,117]. After being stimulated by myocardial infarction, they transdifferentiate into cardiac myofibroblasts and play a fundamental role in the fibrotic healing response. Although the activation of cardiac fibroblasts is crucial for the repair of heart function when intracellular TAGE induce cell death in cardiomyocytes, intracellular TAGE within these cells may inhibit their functions (Figure 5). Since this may prevent the fibrotic healing response, heart dysfunction may occur.

### 8.5. Effects of Extracellular TAGE on Cardiomyocytes and Cardiac Fibroblasts

We previously suggested a role for extracellular TAGE in the vascular system for the promotion of CVD and reported relationships between serum TAGE levels and risk factors for CVD [18,19,20,118,119]. The mechanisms underlying the promotion of CVD by extracellular TAGE or the above risk factors involve vascular disorders. We demonstrated that the activation of the extracellular TAGE-RAGE axis resulted in the generation of intracellular ROS and the subsequent activation of NF-κB in vascular wall cells, which may promote the expression of various atherosclerosis- and inflammation-related genes, thereby contributing to the onset/progression of CVD in DM [23,32].

Since RAGE is expressed in both cardiomyocytes and cardiac fibroblasts, the TAGE-RAGE axis may induce a cytotoxic response in these cells. However, extracellular TAGE do not directly suppress cardiomyocyte pulsation or induce cell death in cardiomyocytes or cardiac fibroblasts (Figure 5).

## 9. Effects of TAGE on Cancer Progression

We previously reported that serum TAGE levels were significantly higher in NBNC-HCC patients than in NASH patients without HCC [16]. The European Prospective Investigation into Cancer and Nutrition (EPIC) also revealed that the risk of rectal cancer after 4 years was elevated in the high serum TAGE group [120]. 

TAGE–RAGE interactions have been shown to alter intracellular signaling cascades in patients with HCC and HSC, and induce angiogenesis, invasion, migration, proliferation, and fibrosis. This cooperation by the TAGE-RAGE axis has been suggested to promote the malignant progression of NASH-related HCC. TAGE induced the expression of CRP in HCC; however, this was attenuated by a pretreatment with anti-RAGE antiserum. The soluble form of RAGE (sRAGE), which functions as a decoy receptor of RAGE, prevented the malignant progression of HCC. Therefore, the TAGE-RAGE axis has potential as a treatment target in NASH and NASH-related HCC [121].

Although their levels are negligible in normal skin, TAGE have been detected in human melanoma tumors. We previously showed that TAGE promoted the growth and migration of human melanoma cells [122]. Furthermore, the formation of tumors by melanoma cell xenografts was prevented in athymic mice through the neutralization of the anti-RAGE antibody. Survival rates were also increased in tumor-bearing mice, and a treatment with an anti-RAGE antibody suppressed spontaneous pulmonary metastases of melanoma. Our findings also revealed that TAGE enhanced the migration and invasion capacities of human lung adenocarcinoma cells [123]. We reported that TAGE increased viable cell numbers and up-regulated RAGE and VEGF mRNA expression in human breast cancer cells [124]. A neutralizing anti-RAGE antibody blocked these increases in viable cell numbers in breast cancer cells. 

Collectively, these findings show the potential roles of TAGE in the growth and invasion of cancer cells by interactions with RAGE.

## 10. Conclusions and Perspectives

Previous studies on AGEs reported that the effects of AGEs were associated with their extracellular binding to RAGE or accumulation in numerous tissues; however, further research on these molecules is needed to clarify the impact of the intracellular generation of TAGE. The in vitro intracellular TAGE molecules that participate in cytotoxic effects in neuroblastoma cells [24,25], hepatocytes [26,27,28], pancreatic cells [29], cardiomyocytes [30], and myoblast cells [31] have been identified. These molecules are produced as intermediates during abnormal glucose and fructose metabolism in the presence of excess GA. TAGE appear to play roles in apoptotic/necrotic events, and thus, have been implicated in cell death and tissue damage. 

Interventional studies have been conducted using TAGE-aptamer and an anti-TAGE antibody. We recently reported that a high-affinity DNA aptamer directed against TAGE (TAGE-aptamer) blocked the progression of DN in an animal model of DM [125]. Furthermore, TAGE-aptamer significantly inhibited the in vivo tumor growth of melanoma cells [126]. TAGE-aptamer also significantly ameliorated neointimal formation after balloon angioplasty in rats [127], and inhibited insulin resistance and adipose tissue remodeling in fructose-fed rats as well as the progression of DN in diabetic mice [128]. We recently reported that an anti-TAGE monoclonal antibody inhibited eye angiogenesis in diabetic mice [PCT/JP2019/34195]. Furthermore, a novel RAGE-blocking antibody improved hind limb perfusion and angiogenesis in diabetic pigs [129]. RAGE-aptamers promoted the regression of experimental DN in diabetic animals, attenuated melanoma growth and metastasis in nude mice, ameliorated renal injury in hypertensive mice, and suppressed renal tubular damage in diabetic mice [130,131,132,133]. Collectively, these findings suggest the potential of the blockade of the TAGE-RAGE axis by TAGE- or RAGE-aptamers and by anti-TAGE or anti-RAGE antibodies as a therapeutic target for DM, CVD, and cancers. 

The novel concept of the “TAGE theory” is expected to open new perspectives for research into numerous other diseases.
